# Associations between Adverse Childhood Experiences and the novel inflammatory marker glycoprotein acetyls in two generations of the Avon Longitudinal Study of Parents and Children birth cohort

**DOI:** 10.1016/j.bbi.2021.11.001

**Published:** 2022-02

**Authors:** Daisy C.P. Crick, Sarah L. Halligan, Laura D. Howe, Rebecca E. Lacey, Golam M. Khandaker, David Burgner, Annie Herbert, Matthew Suderman, Emma L. Anderson, Abigail Fraser

**Affiliations:** a Population Health Sciences, Bristol Medical School, University of Bristol, Bristol, UK; b MRC Integrative Epidemiology Unit, University of Bristol, Bristol, UK; c Department of Psychology, University of Bath, Bath, UK; d Department of Psychiatry and Mental Health, University of Cape Town, South Africa; e Research Department of Epidemiology and Public Health, UCL, London, UK; f Department of Psychiatry, University of Cambridge, Cambridge, UK; g Cambridgeshire and Peterborough NHS Foundation Trust, Cambridge, UK; h Murdoch Children’s Research Institute (MCRI), the Royal Children’s Hospital, Parkville, Victoria, Australia; i Department of Paediatrics, University of Melbourne, Parkville, Victoria, Australia

**Keywords:** Glycoprotein acetyls, Biomarker, Adverse childhood experiences, Inflammation, ALSPAC, Cohort study

## Abstract

**Background:**

Adverse childhood experiences (ACEs) are associated with increased risk of non-communicable diseases in adulthood, potentially mediated by chronic low-grade inflammation. Glycoprotein acetyls (GlycA) is a marker of chronic and cumulative inflammation. We investigated associations between ACEs and GlycA at different ages, in two generations of the population-based Avon Longitudinal Study of Parents and Children (ALSPAC) birth cohort.

**Methods:**

ALSPAC offspring’s total ACE scores were generated for two age periods using prospectively collected data: 0-7y and 0-17y. GlycA was measured using high-resolution proton nuclear magnetic resonance at mean ages 8y, 18y, and 24y. Sample sizes ranged from: n = 5116 (8y) to n = 3085 (24y). ALSPAC mothers (n = 4634) retrospectively reported ACEs experienced before age 18y and GlycA was assessed at mean age 49y. We used multivariable linear regression to estimate associations between ACEs (total ACE score and individual ACEs) and subsequent GlycA in both samples, adjusting for key confounders.

**Results:**

Mean GlycA levels were similar in offspring and mothers and over time. In offspring, there was no evidence that ACEs (total score or individual ACE) were associated with GlycA at age 8y or 18y, or 24y after adjustment for maternal age at birth and parity, maternal marital status, household occupational social class, maternal education, maternal smoking, own ethnicity, sex, and age in months. In mothers, there was evidence of a positive association between the total ACE score and GlycA at age 49y (adjusted mean difference 0.007 mmol/L; 95%CI: 0.003, 0.01). Emotional neglect was the only individual ACE associated with higher GlycA after adjusting for confounders and other ACEs.

**Conclusion:**

Results suggest the association between ACEs and GlycA may emerge in middle age. Future research should explore the extent to which inflammation in adulthood mediates well-documented associations between ACEs and adverse health outcomes in later life.

## Highlights

• Adverse Childhood Experiences positively associate with non-communicable diseases.
• Chronic inflammation may mediate these associations, but evidence is inconsistent.
• Glycoprotein Acetyls is a proposed marker of chronic and cumulative inflammation.
• Total Adverse Childhood Experiences were not associated with GlycA in childhood.
• Total Adverse Childhood Experiences were associated with GlycA in mid-adulthood.

## 1 Introduction

Adverse childhood experiences (ACEs) (Felitti et al., 1998) are experiences such as sexual abuse or substance abuse in the household which require “significant adaptation by the developing child in terms of psychological, social, and neurodevelopmental systems, and which are outside of the normal expected environment” (McLaughlin, 2016). ACEs are common in the UK, and their rates appear to be increasing, with the most prevalent being: maternal mental illness (52.1%), parental divorce/separation (34.7%), or having been verbally abused (22.8%) (Lacey et al.). ACEs tend to co-occur, in other words, exposure to a single ACE also increases the probability of experiencing other ACEs (Lacey et al., 2020, Dong et al., 2004, Sidebotham et al., 2011). Disadvantaged socio-economic background appears to be a key determinant of ACEs and is associated with greater ACE exposure (Lacey et al., 2020, Metzler et al., 2017, Walsh et al., 2019). According to the Family Stress Model (Conger, 1994), poverty places a strain on family relationships and consequently can result in poor parental mental health, suboptimal parenting and interparental conflict (Conger, 2020, Conger et al., 1992).

ACEs are associated with increased risk of non-communicable diseases (NCDs) in adulthood (Hughes et al., 2017, Danese and Tan, 2014, Tsehay et al., 2020, Bennouna-Greene et al., 2011) and ultimately a greater risk of premature mortality (Bellis et al., 2015). It has been postulated that these associations are mediated by dysregulated immune responses, particularly chronic low-grade inflammation (O'Connor et al., 2020, Flouri et al., 2020, Slopen et al., 2013). Childhood is a sensitive period where experiences can alter the biology of the individual (Franceschi and Campisi, 2014, Hertzman, 2012, Viner et al., 2015). Exposure to risk factors during the sensitive period may alter certain physiologic systems permanently, which increases the risk of diseases in adulthood (Joëls and Baram, 2009, Teicher et al., 2016, Tomalski and Johnson, 2010). The immune system could be one such pathway liable to developmental programming because of exposure to stress during early life. As such, it is hypothesised that ACEs may not only trigger a stress response at onset, but also influence long-term stress responses, leading to chronic inflammation (Bucci et al., 2016, Franke, 2014). However, evidence for associations between ACEs and inflammatory biomarkers are inconsistent (Flouri et al., 2020, Russell et al., 2019, Danese et al., 2007, Flouri et al., 2019, Freund et al., 2010, Baumeister, 2016, Kuhlman et al., 2020). Chronic inflammation may play a role in the aetiology of physical (Franceschi and Campisi, 2014, Wang et al., 2002, Lawler et al., 2020) and mental health disorders (Khandaker et al., 2014), many of which are also suggested effects of ACE exposure (Salas, 2019). For example, cardiovascular disease, mood disorders and diabetes are all associated with both higher levels of inflammation and with ACE exposure (Franceschi and Campisi, 2014, Lawler et al., 2020, Salas, 2019, Grant and Dixit, 2013). Therefore chronic and cumulative inflammation is a plausable mechanism for the association between ACEs and subsequent NCDs in later life.

High sensitivity C-Reactive Protein (hsCRP) and interleukin-6 (IL-6) are two of the most commonly used biomarkers in inflammation research (Flouri et al., 2019, Kuhlman et al., 2020, Iob et al., 2019, Lacey, 2020, Chen and Lacey, 2018, Bertone-Johnson et al., 2012). However, hsCRP is an acute-phase reactant (Pepys and Hirschfield, 2003) and largely driven by the acute IL-6 response (Gabay, 2006, Del Giudice and Gangestad, 2018). Both display high intra-individual variability (Connelly et al., 2017) and as a result are likely suboptimal measures of chronic or cumulative inflammation. Glycoprotein Acetyls (GlycA) (Connelly et al., 2017) is a recently described composite inflammatory biomarker that quantifies changes in glycosylation of major plasma proteins (Otvos et al., 20152015). Unlike hsCRP or IL-6 levels, GlycA levels are relatively stable over many years and are not as affected by short-term inflammatory changes (Connelly et al., 2017). Consequently, GlycA may be more reflective of chronic inflammation than acute phase reactants.

Here we investigated the association of ACEs with GlycA in a prospective UK birth cohort: the Avon Longitudinal Study of Parents and Children (ALSPAC) (Boyd et al., 20132013, Fraser et al., 2013, Northstone, 2019), using data on both the index participants in childhood and their mothers. To test associations over the life-course we used GlycA data measured at ages 8y, 18y and 24y in offspring and at the mean age of 49y in mothers. We hypothesise that increased exposure to ACEs will be associated with higher GlycA levels.

## 2 Methods

ALSPAC recruited a total of 14,541 pregnant women residing in the former county of Avon; in South-Western England. The women were recruited if they had an expected delivery date between 1st April 1991 and 31st December 1992. When the oldest children were approximately 7 years of age, an attempt was made to bolster the initial sample with eligible cases who had failed to join the study originally. The total sample size for analyses using any data collected after the age of seven is therefore 15,454 pregnancies, resulting in 15,589 foetuses. Of these, 14,901 were alive at 1 year of age. The offspring, their mothers and the mother’s partners are regularly followed up. The ALSPAC website contains details of all the data that is available through a fully searchable data dictionary and variable search tool http://www.bristol.ac.uk/alspac/researchers/our-data/. Data involving individuals over the age of 22y are collected and managed using REDCap electronic data capture tools, hosted at the University of Bristol (Harris et al., 2009, Harris, 2019). Informed consent for the use of data collected via questionnaires and clinics was obtained from participants following the recommendations of the ALSPAC Ethics and Law Committee at the time. Ethical approval for the collection of biological samples was obtained from the ALSPAC Ethics and Law Committee and the Local Research Ethics Committees. Consent for biological samples has been collected in accordance with the Human Tissue Act (2004).

Offspring were included in the current study if they had at least one measure of GlycA at mean ages 8y, 18y or 24y and the derivation of ACEs was restricted to offspring who answered at least 10% of all questions related to ACE exposure (541 questions) between 0 and 18y. The participant flowchart for the offspring is presented in Fig. 1 in the supplementary file.

Mothers were included if they had a measure of GlycA at one of two clinic assessments conducted at mean age 47y and mean age 50y and had a measure of at least one ACE exposure. Measures of GlycA from the later clinic were prioritised, but if missing, the earlier measure was used, giving a mean age of 49y in our sample. The participant flowchart for this group is presented in Fig. 2 in the supplementary file.

### 2.1 ACE measures

Derivation of the total ACE score for ALSPAC offspring has been described in detail previously (Houtepen, 2018). In brief, a total of 541 questionnaire items were used to generate the ten individual binary ACEs experienced throughout childhood. These included physical abuse, sexual abuse, emotional abuse, emotional neglect, bullying, substance use in the household, violence between parents, parental mental health problems or suicide, parent convicted of an offence and parental separation. A binary (yes/no) construct for a specific ACE exposure during a certain time-period was created if the individual had answered>50% of questions relating to that ACE, in the specified time-period. Participants who had responded to less than 50% of the questions were coded as missing for that binary construct. Multiple imputation was used to impute missing values of individual ACE-related questions. The individual ACEs were summed to create a total ACE score which ranged from 0 to 10. Separate scores were generated for ACEs experienced between 0 and 7y and between 0 and 16y. For outcomes measured at age 8y, the former measure was used to maintain temporal ordering of exposure and outcome variables. For outcomes measured at ages 18y and 24y the latter ACE score was used to capture the fullest possible measure of exposure to ACEs.

Mothers retrospectively reported their exposure to ACEs in several questionnaires administered at enrolment into the study, throughout their pregnancy (from 12 weeks gestation) and postnatally (up to 33 months postnatally). These questions were used to create binary variables for physical abuse, sexual abuse, emotional abuse, emotional neglect, substance use in the household, violence between parents, parental mental health problems and parental separation (see Table 1 in the supplementary file for variables used to create each ACE and their prevalence). Any affirmative to experiencing an ACE across the different questionnaires was prioritised and negated contradictory or missing answers in previous or future questions. There were no questions pertaining to bullying and due to a large number of missing, the ACE relating to a parental conviction was omitted. Therefore, the mother’s ACE score ranged from a minimum of 0 to a potential maximum of 8.

### 2.2 GlycA

Assays for GlycA were performed using high-throughput proton (1H) Nuclear Magnetic Resonance (NMR) on plasma samples (Nightingale Health, Helsinki, Finland). Details regarding sample processing, NMR analysis, and data processing have been detailed elsewhere (Soininen et al., 2009, Inouye, 2010, Kettunen et al., 2012). All blood samples except those collected from offspring at age 8y were fasted for at least 8 h. The distribution of GlycA was checked using histograms and given that they appeared to be normal, the data was not transformed.

### 2.3 Other variables

Mothers reported their own and their fathers’ highest education qualification (degree or above, A level or below) and this was used as a proxy for offspring and own childhood socio-economic position (SEP), respectively (Galobardes et al., 2007). Mothers also reported their own, their partner’s and their parents’ occupations, with respondents asked to provide a job title or summary and indication of their employment seniority. Their responses were then computerised into text strings for coding and finally categorised as manual or non-manual according to the Registrar General’s Social Classes. Each household was assigned the highest reported occupation position held. Household occupational social class and marital status (self-reported during pregnancy) were used as additional indicators of SEP. Mothers reported their smoking pattern during pregnancy and were classified as a “smoker” if they reported smoking in all the trimesters, as a “temporary smoker” if they reported smoking in one of the trimesters and as a “never smoking” if they did not report smoking at all. Mothers and offspring’s ethnicity were reported by mothers during pregnancy and was coded as white or non-white, the mother’s age and parity was also reported during pregnancy. Age of the offspring at blood draw was recorded in months and mothers’ age at blood draw was reported in years.

Measures of maternal and offspring body mass index (BMI) calculated at clinic assessments were used in imputation models. Weight was measured with the use of Tanita scales to the nearest 0.1 kg and height was measured using a Haroenden standiometer to the nearest 0.1 cm. BMI was calculated as weight in kg divided by height in meters squared. As both childhood and adult BMI may be on the causal pathway from ACE to GlycA (i.e., acting as a potential mediator), it was not included as a confounder in analyses.

### 2.4 Statistical analysis

Multivariable linear regression was used to examine associations between the total ACE score, each of the individual ACEs (present/absent), and subsequent GlycA levels measured at 8y, 18y and 24y in offspring and at 49y in mothers. We used the ACE score as a continuum, combining scores of 6 + due to limited numbers (Table 2 of the supplementary file). We tested for departure from linearity using the likelihood ratio test and no evidence for this was found (all *p*-values for non-linearity > 0.12). Therefore, we used the ACE score as a continuum. For both offspring and mothers, we estimated the unadjusted association of the total ACE scores and each individual ACE with GlycA (model 1) and adjusted for potential confounders (model 2). We additionally adjusted individual ACEs, for covariates and other ACEs (model 3). It is important to note that given that ACEs tend to cluster (Lacey et al.) model 3 could suffer over-adjustment. This means that, by adjusting for the individual ACEs, we may bias the results towards the null and thus not detect the individual effects of the ACE of interest. There was no evidence of sex differences in the association between total ACE scores and GlycA at any age in the offspring (all *p*-values for sex interaction > 0.21) and therefore analyses were pooled. Finally, to explore whether there were any systematic differences between those who consented to give blood and those who did not, we investigated all characteristics listed in Table 6 of the supplementary file in the observed data. We found no systematic differences between the two groups.

### 2.5 Missing data

We used multiple imputation (MI) for missing offspring exposures and covariable data and created imputed datasets for two samples: 1) including offspring with data on GlycA at age 8y (N = 5116) and 2) including offspring with data on GlycA at either age 18y or 24y (N = 4377). In the second sample, if GlycA was measured at one of the 18y or 24y time points, we imputed the other measurement; participants were excluded if they had neither measure. Offspring had to have answered > 10% of ACE questions to be eligible for inclusion. The variables used in the imputation model are listed Table 3 of the supplementary file. Data for males and females were imputed separately and then appended. For both males and females, 20 imputed datasets were created using the *mice* package version 2.46.0 in R3.3.1 with 20 iterations per dataset.

We imputed confounder and exposure data for 4634 mothers who had a measure of GlycA at one of the two timepoints and whom had at least one ACE measure. We used 38 variables in the imputation model, 21 of which were auxiliary variables (listed in Table 3 of the supplementary file). We imputed 40 datasets with 10 iterations per dataset. Iterations were set to the maximum amount and the imputation was allowed to run until the data converged. The imputation was run in Stata version 16.1 (Stata Corp, College Station, TX) using the *mi impute chained* command and estimates were then combined through Rubin's rules (Rubin and Schenker, 1991, Sterne et al., 2009) using the command *mi estimate*.

We assumed that data are Missing at Random, i.e., potential systematic differences between the missing and observed values. Therefore, for both the offspring and mothers’ imputation models, we included all exposures, confounders and outcome variables, as well as auxiliary variables to help inform the imputation (Bhaskaran and Smeeth, 2014).

## 3 Results

### 3.1 Distributions of GlycA in ALSPAC mothers and offspring

Mean GlycA in the offspring females was 1.25 mmol/L (SE: 0.003) at age 8y, 1.24 mmol/L (0.003) at age 18y, and 1.24 mmol/L (0.004) at age 24y. Mean GlycA in the offspring males was 1.21 mmol/L (SE: 0.003) at age 8y, 1.18 mmol/L (0.003) at age 18y, and 1.23 mmol/L (0.005) at age 24y. Mean GlycA in the mothers was 1.26 mmol/L (0.003) at age 49y.

Mean GlycA by individual ACE are presented in Table 4 of the supplementary file. Mean GlycA and standard deviation for the complete case data by age and sex is presented in Table 5 of the supplementary file.

Distributions of offspring’s observed and imputed characteristics, other than ACEs at ages 8y, 18y and 24y are presented in supplementary Tables 6 and 7 respectively and in supplementary Table 8 for mothers at age 49y. Consistent with the social patterning of attrition (Howe et al., 2013), ACE medians and interquartile ranges were higher in the imputed data compared to the observed data in the offspring. In the mothers the median total ACE score (median = 1) was consistent between the complete case and imputed datasets. However, the interquartile range spanned 0–1 in the observed data and 0–2 in the imputed data. The distribution of the individual ACEs for both the observed and imputed data can be found in the supplementary file, Table 9 (8y), Table 10 (18y and 24y) and Table 11 (49y).

### 3.2 Association between ACE and GlycA in offspring

The total ACE score was not associated with GlycA measured at ages 8y or 18y in the unadjusted or adjusted models. The total ACE score was associated with GlycA measured at 24y in the unadjusted model, but this attenuated to the null after adjusting for covariates. At age 8y, there were no associations with individual ACEs before or after adjusting for covariates or when adjusting for covariates and other ACEs (Table 1). At age 18y, there were no associations with individual ACEs except for parental separation in the unadjusted model, but this attenuated to the null when adjusting for covariates and when adjusting for covariates and other ACEs (Table 2).

**Table 1:** Associations between ACE and GlycA at 8 years in Offspring.*

Variable	Unadjusted			Adjusted A			Adjusted B		
	Mean difference in GlycA(mmol/L)	95% CI	*p*	Mean difference in GlycA(mmol/L)	95% CI	*p*	Mean difference in GlycA(mmol/L)	95% CI	*p*
Total Ace Score^**^	−0.0003	−0.003, 0.002	0.82	−0.001	−0.004, 0.002	0.50			
Physical abuse	−0.001	−0.01, 0.01	0.87	−0.003	−0.01, 0.01	0.73	−0.002	−0.01, 0.01	0.74
Sexual abuse	0.001	−0.02, 0.02	0.89	−0.01	−0.03, 0.02	0.63	−0.005	−0.03, 0.02	0.66
Emotional abuse	0.002	−0.01, 0.01	0.70	0.003	−0.01, 0.01	0.58	0.01	−0.005, 0.02	0.22
Emotional neglect	−0.0004	−0.01, 0.01	0.94	0.0004	−0.01, 0.01	0.94	0.001	−0.01, 0.01	0.93
Bullying	0.001	−0.01, 0.01	0.79	0.004	−0.01, 0.01	0.42	0.004	−0.01, 0.01	0.41
Substance use in household	−0.0001	−0.01, 0.01	0.99	−0.003	−0.02, 0.01	0.62	−0.002	−0.02, 0.01	0.78
Violence between parents	−0.01	−0.02, 0.01	0.30	−0.01	−0.02, 0.003	0.15	−0.01	−0.02, 0.004	0.15
Parental mental health problems/Suicide	−0.001	−0.01, 0.01	0.74	−0.003	−0.01, 0.01	0.48	−0.003	−0.01, 0.01	0.58
Parent convicted of an offence	−0.003	−0.02, 0.01	0.70	−0.01	−0.02, 0.01	0.43	−0.01	−0.02, 0.01	0.51
Parental separation	0.002	−0.01, 0.01	0.68	−0.001	−0.01, 0.01	0.84	0.0003	−0.01, 0.01	0.95

**Adjusted A.** Adjusted for: Maternal age at birth and parity, maternal marital status, household occupational social class, maternal education, maternal smoking, own ethnicity, sex, and age in months. **Adjusted B.** Adjusted for: Maternal age at birth and parity, maternal marital status, household occupational social class, maternal education, maternal smoking, own ethnicity, sex, and age in months and all other ACEs.

* ACEs score is for the timepoint 0−7y.

** Mean difference of GlycA in mmol/L per one additional ACE.

Bold values = P values <0.05

**Table 2:** Associations between ACE and GlycA at 18 years in Offspring.*

Variable	Unadjusted			Adjusted A			Adjusted B		
	Mean difference in GlycA (mmol/L)	95% CI	*p*	Mean difference in GlycA (mmol/L)	95% CI	*p*	Mean difference in GlycA (mmol/L)	95% CI	*p*
Total Ace Score^**^	0.002	−0.0003, 0.01	0.08	0.001	−0.003, 0.004	0.74			
Physical abuse	0.001	−0.01, 0.01	0.80	0.0001	−0.01, 0.01	0.991	0.0003	−0.01, 0.01	0.97
Sexual abuse	0.02	−0.01, 0.04	0.06	0.02	−0.01, 0.04	0.14	0.02	−0.01, 0.04	0.15
Emotional abuse	−0.00001	−0.01, 0.01	0.998	−0.002	−0.01, 0.01	0.77	−0.004	−0.02, 0.01	0.55
Emotional neglect	0.003	−0.01, 0.02	0.57	0.0002	−0.01, 0.01	0.98	0.0002	−0.01, 0.01	0.98
Bullying	−0.001	−0.01, 0.01	0.88	−0.003	−0.01, 0.01	0.66	−0.003	−0.01, 0.01	0.66
Substance use in household	0.005	−0.01, 0.02	0.56	−0.001	−0.02, 0.02	0.92	−0.002	−0.02, 0.02	0.85
Violence between parents	0.01	−0.01, 0.02	0.23	0.002	−0.01, 0.02	0.72	0.002	−0.01, 0.02	0.81
Parental mental health problems/Suicide	0.01	−0.004, 0.02	0.23	0.004	−0.01, 0.02	0.50	0.004	−0.01, 0.02	0.54
Parent convicted of an offence	0.001	−0.02, 0.02	0.14	−0.003	−0.02, 0.02	0.80	−0.003	−0.02, 0.02	0.78
Parental separation	0.02	0.003, 0.03	0.02	0.01	−0.01, 0.02	0.28	0.01	−0.01, 0.02	0.29

**Adjusted A.** Adjusted for: Maternal age at birth and parity, maternal marital status, household occupational social class, maternal education, maternal smoking, own ethnicity, sex, and age in months. **Adjusted B.** Adjusted for: Maternal age at birth and parity, maternal marital status, household occupational social class, maternal education, maternal smoking, own ethnicity, sex, and age in months and all other ACEs.

* ACE Score is for the timepoint 0–17y.

** Mean difference of GlycA per one additional ACE.

Bold values = P values <0.05.

Associations between ACE scores and GlycA at age 24 are presented in Table 3. The total ACE score was positively associated with GlycA in the unadjusted model (mean difference in GlycA per unit increase in ACE = 0.006 mmol/L, 95% CI: 0.002, 0.01) but this association attenuated to the null after adjustment (0.002; −0.002, 0.01). In the unadjusted model bullying, parental mental health/suicide and parental separation were each associated with higher GlycA. However, after adjusting for covariates and adjusting for covariates and other ACEs, the associations attenuated to the null.

**Table 3:** Associations between ACE and GlycA at 24 years in Offspring.*

Variable	Unadjusted			Adjusted A			Adjusted B		
	Mean difference in GlycA (mmol/L)	95% CI	*p*	Mean difference in GlycA (mmol/L)	95% CI	*p*	Mean difference in GlycA (mmol/L)	95% CI	*p*
Total Ace Score^**^	0.006	0.002, 0.01	0.003	0.002	−0.002, 0.01	0.29			
Physical abuse	0.01	−0.01, 0.02	0.44	0.002	−0.01, 0.02	0.80	0.0004	−0.02, 0.02	0.96
Sexual abuse	0.03	−0.001, 0.05	0.06	0.02	−0.01, 0.04	0.23	0.02	−0.01, 0.04	0.27
Emotional abuse	0.01	−0.01, 0.02	0.19	0.01	−0.01, 0.02	0.50	0.004	−0.01, 0.02	0.65
Emotional neglect	0.003	− 0.02, 0.02	0.74	−0.001	−0.02, 0.02	0.88	−0.002	−0.02, 0.02	0.79
Bullying	0.02	0.0004, 0.04	0.03	0.01	−0.002, 0.03	0.08	0.01	−0.002, 0.03	0.08
Substance use in household	0.001	−0.02, 0.02	0.96	−0.02	−0.04, 0.01	0.16	−0.02	−0.04, 0.003	0.09
Violence between parents	0.01	−0.01, 0.03	0.54	−0.005	−0.02, 0.02	0.65	−0.01	−0.03, 0.01	0.44
Parental mental health problems/Suicide	0.02	0.002, 0.03	0.02	0.01	−0.004, 0.02	0.19	0.01	−0.01, 0.02	0.27
Parent convicted of an offence	0.02	−0.01, 0.04	0.24	0.01	−0.02, −0.03	0.61	0.01	−0.02, 0.03	0.67
Parental Separation	0.03	0.01, 0.04	0.002	0.01	−0.005, 0.03	0.13	0.01	−0.01, 0.03	0.17

**Adjusted A.** Adjusted for: Maternal age at birth and parity, maternal marital status, household occupational social class, maternal education, maternal smoking, own ethnicity, sex, and age in months. **Adjusted B.** Adjusted for: Maternal age at birth and parity, maternal marital status, household occupational social class, maternal education, maternal smoking, own ethnicity, sex, and age in months and all other ACEs.

* ACE Score is from the timepoint 0–17y.

** Mean difference of GlycA per one additional ACE.

### 3.3 Associations between ACEs and GlycA in mothers

Associations between the ACE score, individual ACEs and GlycA are presented in Table 4. There was evidence of a positive association between the total ACE score and GlycA in the unadjusted (mean difference per one additional ACE = 0.006, 95% CI: 0.003, 0.01) and adjusted models (0.007; 0.003, 0.01). Of the individual ACEs, physical abuse, sexual abuse, emotional neglect, and violence between parents were all associated with higher GlycA in the unadjusted and adjusted model. After mutually adjusting for covariates and other ACEs, only the association between emotional neglect and higher GlycA remained. Evidence of an inverse association between substance abuse in the home and GlycA also appeared after adjusting for covariates and all other ACEs.

**Table 4:** Associations between ACE and GlycA at 49 years in Mothers.*

Variable	Unadjusted			Adjusted A			Adjusted B		
	Mean difference in GlycA (mmol/L)	95% CI	*p*	Mean difference in GlycA (mmol/L)	95% CI	*p*	Mean difference in GlycA (mmol/L)	95% CI	*p*
Total ACE score*	0.006	0.003, 0.01	0.001	0.007	0.003, 0.01	0.003			
Physical Abuse	0.04	0.02, 0.06	0.001	0.04	0.01, 0.06	0.004	0.02	−0.004, 0.05	0.14
Sexual Abuse	0.02	0.004, 0.04	0.01	0.02	0.01, 0.04	0.01	0.02	−0.001, 0.03	0.06
Emotional abuse	0.01	−0.005, 0.03	0.14	0.02	−0.004, 0.04	0.15	−0.01	−0.03, 0.01	0.45
Emotional neglect	0.02	0.01, 0.04	0.00	0.03	0.01, 0.04	0.001	0.02	0.004, 0.03	0.03
Substance use in household	−0.01	−0.03, 0.01	0.43	−0.01	−0.03, 0.01	0.29	−0.02	−0.04, −0.0003	0.03
Violence between parents	0.02	0.01, 0.04	0.01	0.02	0.01, 0.04	0.02	0.02	−0.004, 0.04	0.15
Parental mental health problems	0.01	−0.01, 0.02	0.27	0.01	−0.01, 0.02	0.35	0.002	−0.01, 0.01	0.79
Parental separation	0.01	−0.01, 0.02	0.38	0.01	−0.01, 0.02	0.41	−0.003	−0.02, 0.01	0.83

**Adjusted A.** Adjusted for: Mother’s father’s highest education qualification, mother’s parent’s social economic position, own ethnicity, and age in years. **Adjusted B.** Adjusted for: Mother’s father’s highest education qualification, mother’s parent’s social economic position, own ethnicity and age in years and all other ACEs.

* ACE Score is for the timepoint 0–17y.

** Mean difference of GlycA per one additional ACE.

## 4 Sensitivity analysis

A complete case analysis in the offspring found no associations between total ACE scores and GlycA at ages 8y, 18y and 24y in either the unadjusted or adjusted models. In the mothers, a small positive association was found in both the unadjusted model, and this attenuated to the null after adjusting for covariates. These results are presented in the supplementary file, Table 12.

## 5 Discussion

A greater burden of ACEs was associated with higher GlycA in mid-adulthood (age 49y). However, in a different but related cohort, we did not find an association between ACE scores and GlycA in childhood (ages 8y and 18y) or early adulthood (age 24y). Further, the effect sizes of ACE scores on GlycA and individual ACEs on GlycA at 49y are small, e.g. 0.007 mmol/L higher GlycA at age 49, per additional ACE exposure. Moreover, whilst some studies have reported associations between GlycA and health outcomes such as cardiovascular mortality, liver and kidney disease (Kettunen et al., 2018), cancer (Chandler et al., 2016), type 2 diabetes mellitus (Connelly et al., 2016) and all-cause mortality (Fischer et al., 2014, Duprez et al., 20162016) whether these reflect causal effects or not, remains to be determined.

Our findings suggest that dysregulated inflammatory responses, potentially a consequence of ACEs, emerge in mid-adulthood. This association may be driven by behavioural mechanisms as ACEs are positively associated with the likelihood of engaging in risk-taking behaviours (Wiehn et al., 2018, Tucker et al., 2021). For example, maltreatment (which comprised of emotional abuse, physical abuse, sexual abuse and physical neglect) was associated with higher alcohol consumption, a known risk factor for a proinflammatory phenotype (Goldstein et al., 2010). An association between ACEs and inflammation was not evident at 8ys, 18ys or 24y which may be because pro-inflammatory behaviours have not yet been adopted or have not yet had a detectable effect by current biomarkers.

A second explanation is that the association between ACE exposure and inflammatory biomarkers may be physiological. The Hypothalamic-Pituitary-Adrenal (HPA) axis is activated under stress, stimulating the release of “survival hormones”, some of which are inflammatory (Tarullo and Gunnar, 2006). This activation can occur both in childhood and adulthood (Baumeister et al., 2014) and it not only modulates the stress response and inflammatory activity, but is also modulated by inflammatory processes (Bellavance and Rivest, 2014, Pace et al., 2007, Miller et al., 2009, Zunszain et al., 2011). A wealth of evidence links ACEs to chronic HPA axis dysfunction in mid-adulthood (Labonté et al., 2012, McGowan et al., 2009), which may contribute to an observed increase in GlycA and subsequently to the development of inflammatory diseases.

**Fig. 1:**
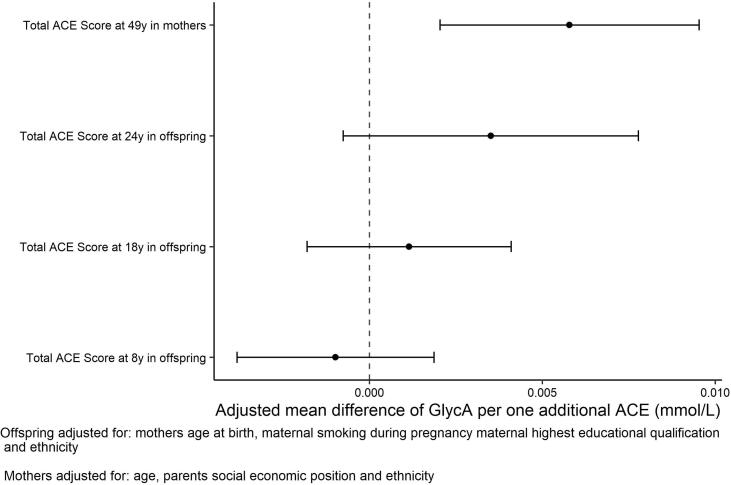
Mean difference in GlycA (mmol/L) per one additional point on the ACE score in the imputed offspring and mothers.

We found differences between individual ACEs driving the association with GlycA between the two generations. In the offspring sample, the only individual ACE associated with GlycA at mean age 24y, after adjusting for confounders was bullying, but this attenuated to the null after mutually adjusting for other ACEs. This is unsurprising given that ACEs co-occur (Bethell et al., 2017). In the mothers, the individual ACEs that maintained an association after adjustment for covariates were physical abuse, sexual abuse and emotional neglect. After mutual adjustment, only emotional neglect remained associated with GlycA and an inverse association between substance-use in the household and GlycA became evident. The increasing magnitude of the ACE-GlycA association over the life course as shown in Fig. 1 suggests that an effect of ACEs on GlycA may emerge over time which could explain the increased number of associations between individual ACEs and GlycA that are apparent in the 49y cohort.

### 5.1 Comparison of results to existing literature

Our observations add to an extant literature that has predominantly focused on markers of acute inflammatory responses and which has produced inconsistent results. Research using the ALSPAC dataset has reported positive associations between parental separation/divorce and IL-6 at age 9y and negative associations between parental alcohol problems, parental metal health problems, parental convictions, emotional abuse, and IL-6 at age 9y (Lacey, 2020). Further research involving ALSPAC has provided evidence that the exposure to adverse life events between 1y and 9y was associated with increased levels of IL-6 at age 9y, which subsequently associated with internalising symptoms at ages 9-11y. However, there was no evidence of a similar association with CRP (Flouri et al., 2019).

Meta-analyses have also aimed to establish whether early-life adversity contributes to potentially pathogenic pro-inflammatory phenotypes in adult individuals. For example, Baumeister et al. (Baumeister, 2016) conducted a 25-study systematic review and meta-analysis and found that individuals exposed to childhood adversity had elevated peripheral levels of CRP, IL-6 and TNF-alpha in adulthood, compared to individuals who had not experienced such adversity. However, another systematic review and meta-analysis of 27 studies also investigating the association between early life adversity and markers of inflammation in children and adolescents, found no evidence of an associations between early life adversity and CRP or IL-6 (Kuhlman et al., 2020). The studies included in the meta-analysis represented a wide range of global populations and diverse methods and nearly half were prospective, longitudinal studies. Highlighted by these systematic reviews, the majority of studies focus on CRP and IL-6, both of which respond to acute inflammatory changes, and are therefore suboptimal markers of chronic inflammation and this could potentially explain the lack of consensus in this research (Iob et al., 2019).

Focus has now moved to biomarkers which may provide more reliable measures of chronic inflammation. For example, exposure to adverse experiences, stress, and violence during childhood or adolescence was associated with levels of the novel inflammatory biomarker suPAR (soluble urokinase plasminogen activator receptor) at 18y. The study was comprised of 1391 participants from the Environmental Risk Longitudinal Twin Study in the United Kingdom; a nationally representative cohort and found elevated SuPAR levels were apparent even in children who did not have elevated hsCRP or IL-6 levels. Additionally, a recent 2020 Australian-based study investigated the association between adversity exposure and the inflammatory biomarkers GlycA and hsCRP in childhood using two different cohorts (O'Connor et al., 2020). In both cohorts, there was weak, inconsistent evidence between adversity exposure and higher hsCRP. However, the study did find a small consistent positive association between adversity exposure and GlycA levels at 4y and 11-12y.

## 6 Strengths and limitations

The summative property of GlycA provides a broad and integrative profile of multiple inflammatory markers and therefore potentially offers a better reflection of chronic systemic inflammation, compared to other inflammatory biomarkers such as hsCRP. A further strength of our study is the use of a large population-based cohort that captured data on two generations. In offspring, ACE data were collected prospectively and GlycA levels were assessed at multiple ages.

A limitation of this study is that, although ACEs were collected prospectively in the offspring, they were collected retrospectively in the mothers. Further, the individual ACEs bullying, and parent convictions were omitted in the mothers. A second limitation is that like most longitudinal cohorts, our study suffers from attrition which can introduce selection bias and likely attenuate results towards the null. However, we used multiple imputation to minimise any such bias.

Finally, the ALSPAC cohort is predominantly white, and this limits the generalisability of these findings to other ethnic groups (Fraser et al., 2013).

## 7 Conclusion

We found modest positive associations between ACEs and GlycA measured at 49y of age. However, we found no evidence of an association between early life adversity in a related cohort at ages 8y, 18y and 24y. This suggests that the association between ACEs and GlycA may emerge in in midlife. It is worth noting that our results are drawn from two different though related cohorts of mothers and their offspring. ACEs were assessed differently (the mothers did not have information on bullying or parental convictions and reported their own ACE exposure retrospectively in adulthood, whereas, offspring’s ACEs were reported prospectively, first by the mothers and then by the offspring themselves (when they were old enough). Thus, although we show that an association between ACE and GlycA levels emerge with age, we cannot be certain whether the associations in the mothers at 49y are an increasing effect across the life course, a cohort effect, or otherwise. Whether this inflammatory pathway mediates the associations between ACEs and mental and physical diseases in adulthood remains to be determined.

## Declaration of Competing Interest

The authors declare that they have no known competing financial interests or personal relationships that could have appeared to influence the work reported in this paper.
