# Supplementary material for: Associations between Adverse Childhood Experiences and the novel inflammatory marker glycoprotein acetyls in two generations of the Avon Longitudinal Study of Parents and Children birth cohort
Source: Brain Behav Immun. 2022 Feb;100:112–20. doi: 10.1016/j.bbi.2021.11.001 (PMC8791601; doi:10.1016/j.bbi.2021.11.001)
Supplement: Supplementary data 1 [file mmc1.docx]

| ACE | Variables | Yes  N (%) | No  N (%) | N missing (%) |
| --- | --- | --- | --- | --- |
| Physical abuse | Parent physically cruel | 120 (2.59) | 4077 (87.98) | 437 (9.43) |
|  | Physically abused by mother, father or other | 203 (4.38) | 3693 (79.69) | 738 (15.93) |
| Sexual abuse | Forced intercourse | 66 (1.42) | 3848 (83.04) | 720 (15.54) |
|  | Abuse / Fondled | 395 (8.52) | 3519 (75.94) | 720 (15.54) |
|  | Abuse/ Oral sex | 48 (1.04) | 3766 (81.27) | 820 (17.70) |
|  | Abuse/ Saw other masturbate | 131 (2.83) | 3732 (80.54) | 771 (16.64) |
|  | Abuse/ rubbed genitals | 213 (4.60) | 3701 (79.87) | 720 (15.54) |
| Emotional abuse | Parent emotionally cruel | 327 (7.06) | 3870 (83.51) | 437 (9.43) |
| Emotional neglect | Emotionally neglected by parent | 783 (16.90) | 3122 (67.37) | 729 (15.73) |
| Substance Household | Maternal alcohol problems | 100 (2.16) | 4042 (87.22) | 492 (10.62) |
|  | Paternal alcohol problems | 253 (5.46) | 3819 (82.41) | 492 (10.62) |
| Violence between parents | Mother and father relationship violent | 427 (9.21) | 3208 (69.23) | 999 (21.56) |
| Parental mental health problems or suicide | Parental mentally ill | 199 (4.29) | 3998 (86.28) | 437 (9.43) |
|  | Mental illness in the family | 1093 (23.59) | 3099 (66.88) | 442 (9.54) |
|  | Maternal schizophrenic | 11 (0.24) | 4137 (89.27) | 486 (10.49) |
|  | Paternal schizophrenic | 10 (0.22) | 4087 (88.20) | 537 (11.59) |
|  | Maternal depression | 840 (18.13) | 3172 (68.45) | 622 (13.42) |
|  | Paternal depression | 341 (7.36) | 3682 (79.46) | 611 (13.19) |
| Parental separation | Parents separated | 559 (12.06) | 3638 (78.51) | 437 (9.43) |
| Table 1: Prevalence of variables combined to create ACEs in ALSPAC mothers (N=4634) | | | | |

|  | Offspring | | | | Mothers | |
| --- | --- | --- | --- | --- | --- | --- |
|  | 7y | | 18y and 24y | | 49y | |
|  | Observed | Imputed | Observed | Imputed | Observed | Imputed |
| ACE Score | N (%) | Prevalence (%) | N (%) | Prevalence (%) | N (%) | % |
| 0 | 1750 (33.11) | 17.87 | 1331 (31.30) | 17.64 | 2108 (45.49) | 46.73 |
| 1 | 1470 (27.81) | 25.89 | 1214 (28.55) | 24.67 | 1145 (24.71) | 26.75 |
| 2 | 888 (16.80) | 21.68 | 799 (18.79) | 21.09 | 505 (10.90) | 11.83 |
| 3 | 522 (9.88) | 14.66 | 461 (10.84) | 14.38 | 254 (5.48) | 6.42 |
| 4 | 285 (5.39) | 9.85 | 256 (6.02) | 9.63 | 151 (3.26) | 4.02 |
| 5 | 130 (2.46) | 5.79 | 122 (2.87) | 6.23 | 76 (1.64) | 2.09 |
| 6+ | 71 (1.34) | 4.25 | 69 (1.62) | 6.34 | 63 (1.36) | 2.17 |
| Missing | 169 (3.20) |  | 125 (2.87) |  | 332 (7.16) |  |
| Table 2: Prevalence of offspring and mothers in each ACE Score category with ACEs from 6-10 combined into a 6+ group in the observed and imputed data | | | | | | |

| **Offspring** |
| --- |
| Offspring’s Sex |
| Ethnicity |
| Preferred birthweight |
| Gestation at delivery or death |
| Pre-pregnancy weight (kg) |
| BMI or Quetelet index during pregnancy |
| Home ownership of mother during pregnancy |
| Grouped age of mother at delivery |
| Marital status of mother during pregnancy |
| Mother’s highest educational qualification |
| Mother’s Partner’s highest educational qualification |
| Became homeless since pregnant |
| Mother is homeless this pregnancy |
| Difficulty in affording food |
| Difficulty in affording heating |
| Mother’s partner became homeless during pregnancy |
| Mother’s Edinburgh post-natal depression score |
| Partner’s Edinburgh post-natal depression score |
| Mother taking medication for anxiety during pregnancy |
| Mother taking medication for depression during pregnancy |
| Mother taking medication for depression in first three months of birth |
| Mother attempted suicide since pregnant |
| Mother taking medication for anxiety in last three months |
| Mother taking medication for depression in last three months |
| Mother had bulimia |
| Mother had schizophrenia |
| Mother had anorexia nervosa |
| Mother’s partner had bulimia |
| Mother’s partner had schizophrenia |
| Mother’s partner had anorexia nervosa |
| Mother’s partner has drug addiction  Mother’s opinion of neighborhood |
| Mother convicted of an offence since pregnant |
| Mother’s partner convicted of an offence since pregnant |
| Mother separated from partner since pregnant |
| Mother divorced since partner was pregnant |
| Household social class at 18 weeks gestation |
| Mother has no one to share feelings with |
| Mother smoked cannabis in 1-3months of pregnancy |
| Mother smoked cannabis after 3 months of pregnancy |
| Mother had taken hard drugs |
| Mother had drug addiction |
| Partner had taken hard drugs |
| Mother’s partner was emotionally cruel to mother’s children in last year |
| Mother was cruel to own children in last year |
| Father was emotionally cruel to children in last year |
| Degree of difficulty household finds trying to pay the bills |
| Frequency mother takes pills for depression in last two years |
| Mother attempted suicide in last year |
| Mother’s Edinburgh post-natal depression score |
| Mother ever admitted to hospital for psychiatric or mental health problems |
| Father ever admitted to hospital for psychiatric or mental health problems |
| Mother ever had an illness that included hearing voices or seeing things |
| Mother ever had an illness with paranoid delusions or developed unusual thoughts |
| Father ever has an illness with paranoid delusions or developed unusual thoughts |
| Mother ever suffered from schizophrenia |
| Father ever suffered from schizophrenia |
| Mother ever suffered from a manic illness |
| Father ever suffered from a manic illness |
| Frequency father has taken pills for depression in the last two years |
| Father’s opinion of neighborhood as place to live |
| Mother was convicted of an offence in last year |
| Father was convicted of an offence in last year |
| Mother was divorced in last year |
| Mother and partner was separated in last year |
| Mother’s partner was physically cruel to mother’s children in last year |
| Mother was physically cruel to mother in last year |
| Frequency mother took cannabis in last two years |
| Frequency mother took cocaine in last two years |
| Frequency mother took amphetamines, ecstasy or other stimulants in last two years |
| Frequency mother took heroin, methadone, crack or other hard drugs in last two years |
| Mother’s AUDIT total score |
| Frequency father took cannabis in last two years |
| Frequency father took cocaine in last two years |
| Frequency father took amphetamines, ecstasy or other stimulants in last two years |
| Frequency father took heroin, methadone, crack or other hard drugs in last two years |
| Child’s age when mother’s partner used physical force such as pushing or slapping |
| Child’s age when mother’s partner used severe physical force such as punching |
| Child’s age when mother’s partner pressured them into kissing or touching |
| Child’s age when mother’s partner forced them into kissing or touching |
| Child’s age when mother’s partner pressured them into sexual intercourse |
| Child’s age when mother’s partner forced them into sexual intercourse |
| Child’s age when mother’s partner’s behavior made them feel scared or frightened |
| Child has ever been aware of an affected by one ‘parent’ slapping or kicking other ‘parent’ |
| Mother’s partner was physically cruel to respondent in last year |
| Mother smoked tobacco in first three months of pregnancy |
| Mother smoked tobacco in last two weeks |
| Number of cigarettes smoked per day |
| Number of cigarettes smoked per day in last two months of pregnancy |
| BMI at age 7y |
| BMI at age 18y |
| BMI at age 24y |
| GlycA at age 7y |
| GlycA at 18y |
| GlycA at 24y |
| Log CRP at 9y |
| Log CRP at 15y |
| Log CRP at 18y |
| Log CRP at 24y |
| Log IL-6 at 9y |
| Age at 7y |
| Age at 18y |
| Age at 24y |
| Maternal smoking during pregnancy |
| Parity |
| **Mothers** |
| Family got poorer |
| Maternal highest education qualification maternal |
| Paternal highest education qualification |
| Own highest education qualification |
| Parent’s social economic position |
| Control attempted by mother |
| Privacy invaded by mother |
| Felt unwanted by mother |
| Adopted |
| In local authority care |
| Lived in foster-parents home |
| Stayed in children’s home |
| Mother in the house between 0-5yrs |
| Mother in the house between 6-11yrs |
| Mother in the house between 12-16yrs |
| Father in the house between 0-5yrs |
| Father in the house between 6-11yrs |
| Father in the house between 12-16yrs |
| Parents’ relationship frightening |
| Parents’ relationship remote |
| Childhood happy |
| Happy between 0-5yrs |
| Happy between 6-11yrs |
| Happy between 12-16yrs |
| CRP level at clinic 1 |
| CRP level at clinic 2 |
| CRP level at clinic 3 |
| CRP level at clinic 4 |
| GlycA at clinic 1 |
| GlycA at clinic 2 |
| Age at clinic 1 |
| Age at clinic 1 |
| Mother’s age at child’s birth |
| Housing tenure during childhood |
| Parity |
| Smoking in pregnancy |
| Alcohol use |
| Table 3: Auxiliary variables included in the imputation model for both the offspring and the mothers |

|  | 8y group | | 18y group | | 24y group | | 49y group | |
| --- | --- | --- | --- | --- | --- | --- | --- | --- |
| Individual ACE | N | Mean (SD) | N | Mean (SD) | N | Mean (SD) | N | Mean (SD) |
| Physical abuse | 549 | 1.22 (0.13) | 410 | 1.21 (0.14) | 462 | 1.23 (0.18) | 246 | 1.29 (0.17) |
| Sexual abuse | 126 | 1.23 (0.12) | 99 | 1.25 (0.14) | 133 | 1.25 (0.18) | 533 | 1.28 (0.18) |
| Emotional abuse | 744 | 1.23 (0.14) | 437 | 1.21 (0.13) | 464 | 1.23 (0.17) | 329 | 1.28 (0.18) |
| Emotional neglect | 680 | 1.23 (0.14) | 470 | 1.21 (0.13) | 426 | 1.23 (0.18) | 783 | 1.27 (0.18) |
| Bullying | 1080 | 1.23 (0.14) | 725 | 1.21 (0.13) | 663 | 1.24 (0.18) | - | - |
| Substance use in household | 361 | 1.23 (0.13) | 213 | 1.21 (0.14) | 209 | 1.23 (0.17) | 323 | 1.25 (0.17) |
| Violence between parents | 689 | 1.22 (0.13) | 401 | 1.22 (0.14) | 397 | 1.23 (0.17) | 427 | 1.27 (0.17) |
| Parental mental health problems/ Suicide | 1680 | 1.23 (0.13) | 1001 | 1.22 (0.14) | 1013 | 1.23 (0.18) | 1115 | 1.27 (0.17) |
| Parent convicted of an offence | 271 | 1.22 (0.15) | 151 | 1.22 (0.13) | 171 | 1.23 (0.16) | - | - |
| Parental separation | 879 | 1.23 (0.14) | 525 | 1.22 (0.13) | 520 | 1.24 (0.17) | 559 | 1.27 (0.18) |
| Table 4: Mean GlycA in the ACE derived group for the offspring at 8y, 18y and 24y and in the mothers at 49y | | | | | | | | |

| Age | N | Mean GlycA (mmol/L) (SD) |
| --- | --- | --- |
| Females | | |
| 8y | 2487 | 1.25 (0.14) |
| 18y | 1591 | 1.25 (0.14) |
| 24y | 1842 | 1.24 (0.17) |
| Males | | |
| 8y | 2629 | 1.21 (0.13) |
| 18y | 1472 | 1.18 (0.13) |
| 24y | 1243 | 1.23 (0.18) |
| Mothers | | |
| 49y | 4634 | 1.26 (0.17) |
| Table 5: Mean GlycA by age and sex for the complete case data | | |

| Variable | Observed (participants with data for at least 10% of ACE questions) | | |  |  | Imputed data (N=5116) |  |
| --- | --- | --- | --- | --- | --- | --- | --- |
|  | Categories | N | Mean (SE) for continuous variables Number (%) for categorical variables | Median (IQR) | % data imputed | Mean (SE) for continuous variables Number (%) for categorical variables | Median (IQR) |
| Total ACE Score 8y |  | 5116 | - | 1 (0-2) | 0 |  | 2 (1-3) |
| Age at 8y in months |  | 5116 | 90.27 (0.05) |  | 0 | 90.27 (0.05) |  |
| Ethnicity | White | 4539 | 96.64 |  | 8.19 | 93.16 |  |
|  | Non-white | 158 | 3.36 |  |  | 6.85 |  |
| Sex | Male | 2629 | 51.39 |  | 0 | 51.39 |  |
|  | Female | 2487 | 48.61 |  |  | 48.61 |  |
| BMI at age 8y (kg/m^2^) |  | 5083 | 16.17 (0.03) |  | 0.65 | 16.17 (.03) |  |
| Mothers age at child’s birth |  | 4892 | 29.27 (0.07) |  | 4.38 | 29.07(.07) |  |
| Maternal smoking during pregnancy? | Never | 3953 | 82.13 |  | 5.92 | 79.21 |  |
|  | Temporary | 268 | 5.57 |  |  | 5.78 |  |
|  | Throughout | 592 | 12.30 |  |  | 15.00 |  |
| Housing at child’s birth | Rented/other | 787 | 16.49 |  | 6.68 | 20.22 |  |
|  | Mortgaged/owned | 3987 | 83.51 |  |  | 79.78 |  |
| Mother’s educational level | Less than degree | 3935 | 82.60 |  | 6.88 | 82.62 |  |
|  | Degree or above | 829 | 17.040 |  |  | 17.38 |  |
| Mother’s parity | 0 | 2118 | 44.53 |  | 7.04 | 44.36 |  |
|  | 1-3 | 2594 | 54.54 |  |  | 54.59 |  |
|  | 4+ | 44 | 0.94 |  |  | 1.05 |  |
| Mother’s marital status | Never married | 639 | 13.27 |  | 5.86 | 15.65 |  |
|  | 1^st^ marriage | 3650 | 75.79 |  |  | 72.21 |  |
|  | Marriage 2+ | 320 | 6.64 |  |  | 6.42 |  |
|  | Separated/divorced/widowed | 207 | 3.30 |  |  | 5.72 |  |
| Household social economic position | Non-manual | 2337 | 59.02 |  | 22.60 | 55.26 |  |
|  | Manual | 1623 | 40.98 |  |  | 44.73 |  |
| Average GlycA at age 8y (mmol/L) |  | 5116 | 1.23 (0.002) |  | 0 | 1.23 (0.002) |  |
| Table 6: Characteristics of offspring with GlycA measured at the 8y clinic assessment in the imputed and observed data (i.e., without imputation) | | | | | | | |
|  |  |  |  |  |  |  |  |

| Variable | Observed Cases (participants with data for at least 10% of ACE questions) | | |  |  | Imputed data (N=4377) |  |
| --- | --- | --- | --- | --- | --- | --- | --- |
|  | Categories | N | Mean (SE) for continuous variables % for categorical variables | Median (IQR) | % data imputed | Mean (SE) for continuous variables % for categorical variables | Median (IQR) |
| Total ACE score |  | 4254 |  | 1 (0-2) | 2.86 | 2.18 (.03) | 2 (1-3) |
| Age at 18y in months |  | 3780 | 213.41 (0.08) |  | 13.64 | 213.54 (0.08) |  |
| Age at 24y in months |  | 3280 | 293.83 (0.17) |  | 25.06 | 294.08 (0.17) |  |
| Ethnicity (white) | White | 3867 | 95.81 |  | 7.79 | 93.25 |  |
|  | Non-white | 169 | 4.19 |  |  | 6.75 |  |
| Sex (Male) | Male | 1930 | 44.09 |  | 0 | 44.09 |  |
|  | Female | 2447 | 55.91 |  |  | 55.91 |  |
| BMI at 18y (kg/m^2^) |  | 3692 | 22.69 (0.07) |  | 15.65 | 22.75 (0.06) |  |
| BMI at 24y (kg/m^2^) |  | 3244 | 24.78 (0.09) |  | 25.89 | 24.92 (0.08) |  |
| Mothers age at child’s birth |  | 4197 | 29.33 (0.07) |  | 4.11 | 29.23 (0.07) |  |
| Maternal smoking during pregnancy? | Never | 3443 | 83.65 |  | 5.96 | 81.28 |  |
|  | Temporary | 178 | 4.32 |  |  | 4.55 |  |
|  | Throughout | 495 | 12.03 |  |  | 14.17 |  |
| Housing at child’s birth | Rented/other | 625 | 15.29 |  | 6.60 | 18.22 |  |
|  | Mortgaged/owned | 3463 | 84.71 |  |  | 81.78 |  |
| Mother’s educational level | Less than degree | 3278 | 80.30 |  | 6.74 | 80.62 |  |
|  | Degree | 804 | 19.70 |  |  | 19.38 |  |
| Mother’s parity | 0 | 1897 | 46.60 |  | 6.99 | 45.97 |  |
|  | 1-3 | 2142 | 52.62 |  |  | 53.03 |  |
|  | 4-8 | 32 | 0.79 |  |  | 1.00 |  |
| Mother’s marital status | Never married | 531 | 12.87 |  | 5.76 | 15.13 |  |
|  | 1^st^ marriage | 3130 | 75.88 |  |  | 72.89 |  |
|  | Marriage 2+ | 276 | 6.69 |  |  | 6.59 |  |
|  | Separated/divorced/widowed | 188 | 4.56 |  |  | 5.39 |  |
| Household social economic position | Non-manual | 1662 | 56.32 |  | 32.58 | 51.18 |  |
|  | Manual | 1289 | 43.68 |  |  | 48.82 |  |
| GlycA at age 18y (mmol/L) |  | 3063 | 1.21 (0.002) |  | 30.02 | 1.22 (0.002) |  |
| GlycA at age 24y (mmol/L) |  | 3085 | 1.23 (0.003) |  | 29.52 | 1.24 (0.003) |  |
| Table 7: Characteristics of offspring with GlycA measured at the 18y or 24y clinic assessment in the imputed and observed data (i.e., without imputation) | | | | | | | |

| Variable | Observed (Participants with at least a measure of GlycA at clinic 1 or clinic) | | |  |  | Imputed data (N=4634) |  |
| --- | --- | --- | --- | --- | --- | --- | --- |
|  | Categories | N | Mean (SE) for continuous variables Number (%) for categorical variables | Median (IQR) | % data imputed | Mean (SE) for continuous variables % for categorical variables | Median (IQR) |
| Total ACE Score |  | 4302 | - | 1 (0-1) | 7.16 | - | 1(0-2) |
| Age based on GlycA used (years) |  | 4634 | 48.92 (0.07) |  | 0 | 48.92 (0.07) |  |
| Ethnicity | White | 4109 | 97.72 |  | 9.26 | 97.62 |  |
|  | Non-white | 96 | 2.28 |  |  | 2.38 |  |
| BMI at 49y (kg/m^2^) |  | 4612 | 26.67 (0.08) |  | 0.47 | 26.67 (0.08) |  |
| Mothers age at child’s birth |  | 4336 | 29.58 (0.07) |  | 6.43 | 29.61 (0.07) |  |
| Maternal smoking during pregnancy | Never | 3637 | 85.06 |  | 7.73 | 84.91 |  |
|  | Temporary | 192 | 4.49 |  |  | 4.52 |  |
|  | Throughout | 447 | 10.45 |  |  | 10.57 |  |
| Maternal alcohol drinking during pregnancy | Never | 780 | 18.97 |  | 11.29 | 18.98 |  |
|  | Yes | 3331 | 81.03 |  |  | 81.02 |  |
| Housing at child’s birth | Rented/other | 480 | 12.34 |  | 14.33 | 12.34 |  |
|  | Mortgaged/ owned | 3480 | 87.66 |  |  | 87.66 |  |
| Mother’s educational level | Less than degree | 3419 | 81.02 |  | 8.93 | 81.12 |  |
|  | Degree or above | 801 | 18.98 |  |  | 18.88 |  |
| Mother’s father’s highest educational level | O level or below | 2167 | 70.13 |  | 33.32 | 72.31 |  |
|  | A level or above | 923 | 29.87 |  |  | 27.69 |  |
| Mother’s parent’s social economic position | Manual | 925 | 40.13 |  | 50.26 | 41.31 |  |
|  | Non-manual | 1380 | 59.83 |  |  | 58.69 |  |
| GlycA at latest cohort |  | 4634 | 1.26 (0.002) |  | 0 | 1.26 (0.002) |  |
| Table 8: Characteristics of mothers with GlycA measured at mean age 49y in the imputed and observed data (i.e., without imputation) | | | | | | | |

| Variable | ACE-derived group (participants with data for at least 10% of ACE questions) | | |  | Imputed data (N=5116) |
| --- | --- | --- | --- | --- | --- |
|  | N | Categories | % | % data imputed | % |
| Physical abuse | 3530 | Yes | 15.55 | 31.00 | 21.33 |
|  |  | No | 84.45 |  | 78.66 |
| Sexual abuse | 4597 | Yes | 2.74 | 10.14 | 5.70 |
|  |  | No | 97.26 |  | 94.30 |
| Emotional abuse | 3707 | Yes | 20.07 | 27.54 | 26.35 |
|  |  | No | 79.93 |  | 73.65 |
| Emotional neglect | 3568 | Yes | 19.06 | 30.26 | 20.92 |
|  |  | No | 80.94 |  | 79.08 |
| Bullying | 4375 | Yes | 24.69 | 14.48 | 25.05 |
|  |  | No | 75.31 |  | 74.95 |
| Violence between parents | 3649 | Yes | 18.88 | 28.67 | 23.09 |
|  |  | No | 81.12 |  | 76.91 |
| Substance abuse in the household | 3919 | Yes | 9.21 | 23.40 | 16.11 |
|  |  | No | 90.79 |  | 83.89 |
| Mental health and suicide problems | 3979 | Yes | 42.22 | 22.22 | 47.54 |
|  |  | No | 57.78 |  | 54.18 |
| Parental conviction | 4021 | Yes | 6.74 | 21.40 | 11.41 |
|  |  | No | 93.26 |  | 88.59 |
| Parental separation | 3594 | Yes | 24.46 | 29.75 | 31.03 |
|  |  | No | 75.54 |  | 68.97 |
| Table 9: Distributions of the individual ACEs in the observed and imputed datasets in the 8y offspring data | | | | | |

| Variable | | ACE-derived group (participants with data for at least 10% of ACE questions) | | |  | Imputed data (N=4377) |
| --- | --- | --- | --- | --- | --- | --- |
|  |  | N | Categories | % | % data imputed | % |
| Physical abuse | | 3250 | Yes | 18.25 | 25.75 | 22.96 |
|  |  |  | No | 81.75 |  | 77.04 |
| Sexual abuse | | 3872 | Yes | 4.16 | 11.54 | 6.60 |
|  |  |  | No | 95.84 |  | 93.40 |
| Emotional abuse | | 3292 | Yes | 19.41 | 24.79 | 23.55 |
|  |  |  | No | 80.59 |  | 76.45 |
| Emotional neglect | | 3512 | Yes | 17.80 | 19.76 | 18.79 |
|  |  |  | No | 82.20 |  | 81.21 |
| Bullying | | 3792 | Yes | 25.47 | 13.37 | 26.25 |
|  |  |  | No | 74.53 |  | 73.75 |
| Violence between parents | | 3135 | Yes | 18.24 | 28.38 | 22.54 |
|  |  |  | No | 81.76 |  | 77.46 |
| Substance abuse in the household | | 3347 | Yes | 9.08 | 23.53 | 15.16 |
|  |  |  | No | 90.92 |  | 84.84 |
| Mental health and suicide problems | | 3442 | Yes | 41.55 | 21.36 | 45.33 |
|  |  |  | No | 58.45 |  | 54.67 |
| Parental conviction | | 3443 | Yes | 6.51 | 21.34 | 9.96 |
|  |  |  | No | 93.49 |  | 90.04 |
| Parental separation | | 3173 | Yes | 23.89 | 27.51 | 30.72 |
|  |  |  | No | 76.11 |  | 69.28 |
| Table 10: Distributions of the individual ACEs in the observed and imputed datasets in the 18y-24y offspring data | | | | | | |
|  |  |  |  |  |  |  |

| Variable | Observed data (Participants with at least a measure of GlycA at clinic 1 or clinic 2) | | |  | Imputed data (N=4634) |
| --- | --- | --- | --- | --- | --- |
|  | N | Categories | % | % data imputed | % |
| Physical abuse | 4269 | Yes | 5.31 | 7.88 | 6.46 |
|  |  | No | 86.81 |  | 93.54 |
| Sexual abuse | 3914 | Yes | 11.50 | 15.54 | 13.74 |
|  |  | No | 72.96 |  | 86.26 |
| Emotional abuse | 4197 | Yes | 7.06 | 9.43 | 8.03 |
|  |  | No | 83.51 |  | 91.97 |
| Emotional neglect | 3905 | Yes | 16.90 | 15.73 | 20.60 |
|  |  | No | 67.37 |  | 79.40 |
| Violence between parents | 3635 | Yes | 9.21 | 21.56 | 12.81 |
|  |  | No | 69.23 |  | 87.19 |
| Substance abuse in the household | 4046 | Yes | 6.97 | 12.69 | 8.49 |
|  |  | No | 80.34 |  | 91.50 |
| Mental health and suicide problems | 4284 | Yes | 24.06 | 7.55 | 26.72 |
|  |  | No | 68.39 |  | 73.30 |
| Parental separation | 4197 | Yes | 12.06 | 9.43 | 26.70 |
|  |  | No | 78.51 |  | 86.45 |
| Table 11: Distributions of the individual ACEs in the observed and imputed datasets in the 49y mother data | | | | | |

|  | | Unadjusted | |  |  | Adjusted | |  |  |
| --- | --- | --- | --- | --- | --- | --- | --- | --- | --- |
|  | | Mean difference | 95% CI | ***p*** | N | Mean difference | 95% CI | ***p*** | N |
|  | Age 7 years | | | | | | |  |  |
| Total Ace Score | | -0.001 | -0.003, 0.002 | 0.66 | 5116 | -0.001 (0.002) | -0.004, 0.002 | 0.50 | 3811 |
|  | Age 17 years | | | | | | |  |  |
| Total Ace Score | | 0.001 | -0.002, 0.005 | 0.41 | 2978 | 0.001 (0.002) | - 0.003, 0.01 | 0.56 | 1991 |
|  | Age 24 years | | | | | | |  |  |
| Total Ace Score | | 0.001 | -0.003, 0.01 | 0.59 | 3007 | -0.001 | -0.01, 0.004 | 0.82 | 2064 |
| Age 49 years | |  |  |  |  |  |  |  |  |
| Total Ace Score | | 0.01 | 0.001, 0.01 | 0.01 | 4302 | 0.01 | -0.0005, 0.01 | 0.07 | 1728 |
| Table 12: Associations between total ACE score and GlycA in the complete data at 8y, 18y and 24y in children and 49y in mothers | | | | | | | | | |


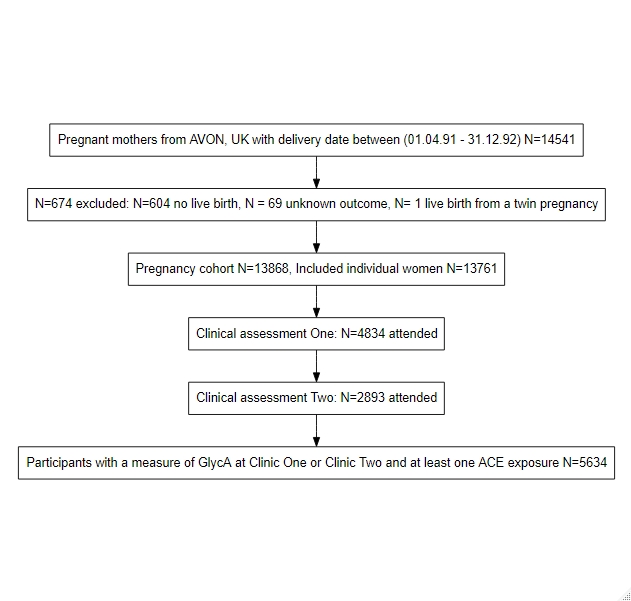


Figure 2: Inclusion criteria for mothers


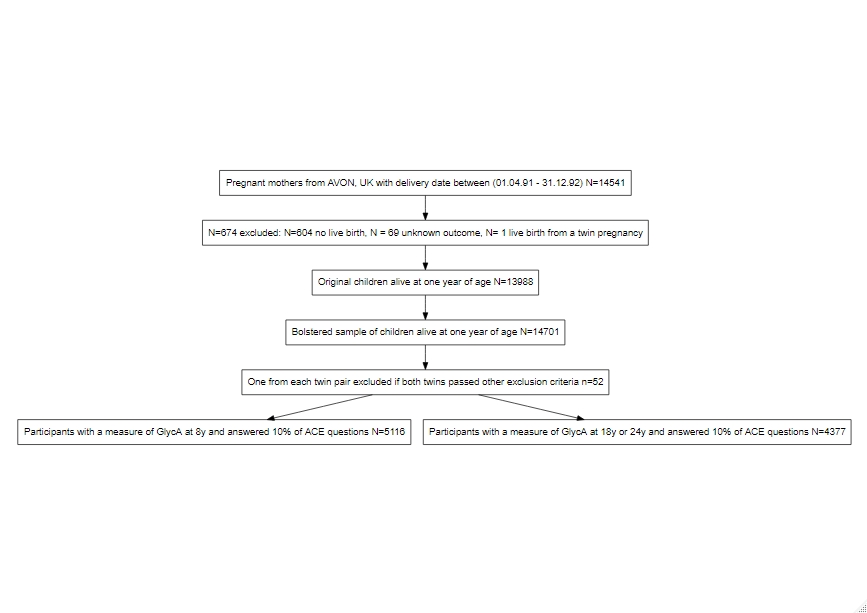


Figure 1: Inclusion criteria for Offspring
